# Functional characteristics of Th1, Th17, and ex-Th17 cells in EAE revealed by intravital two-photon microscopy

**DOI:** 10.1186/s12974-020-02021-x

**Published:** 2020-11-26

**Authors:** Julia Loos, Samantha Schmaul, Theresa Marie Noll, Magdalena Paterka, Miriam Schillner, Julian T. Löffel, Frauke Zipp, Stefan Bittner

**Affiliations:** Department of Neurology, Focus Program Translational Neuroscience (FTN) and Immunotherapy (FZI), Rhine Main Neuroscience Network (rmn2), University Medical Center of the Johannes Gutenberg-University of Mainz, Mainz, Germany

**Keywords:** EAE, Th1 cells, Th17 cells, Two-photon microscopy

## Abstract

**Background:**

T helper (Th) 17 cells are a highly plastic subset of T cells, which in the context of neuroinflammation, are able to acquire pathogenic features originally attributed to Th1 cells (resulting in so called ex-Th17 cells). Thus, a strict separation between the two T cell subsets in the context of experimental autoimmune encephalomyelitis (EAE) is difficult. High variability in culture and EAE induction protocols contributed to previous conflicting results concerning the differential contribution of Th1 and Th17 cells in EAE. Here, we systematically evaluate the role of different T cell differentiation and transfer protocols for EAE disease development and investigate the functional dynamics of encephalitogenic T cells directly within the inflamed central nervous system (CNS) tissue.

**Methods:**

We compiled the currently used EAE induction protocols reported in literature and investigated the influence of the different Th1 and Th17 differentiation protocols as well as EAE induction protocols on the EAE disease course. Moreover, we assessed the cytokine profile and functional dynamics of both encephalitogenic Th1 and Th17 cells in the inflamed CNS using flow cytometry and intravital two-photon laser scanning microscopy. Lastly, we used astrocyte culture and adoptive transfer EAE to evaluate the impact of Th1 and Th17 cells on astrocyte adhesion molecule expression in vitro and in vivo.

**Results:**

We show that EAE courses are highly dependent on in vitro differentiation and transfer protocols. Moreover, using genetically encoded reporter mice (B6.IL17A-EGFP.acRFP x 2d2/2d2.RFP), we show that the motility of interferon (IFN)γ-producing ex-Th17 cells more closely resembles Th1 cells than Th17 cells in transfer EAE. Mechanistically, IFNγ-producing Th1 cells selectively induce the expression of cellular adhesion molecules I-CAM1 while Th1 as well as ex-Th17 induce V-CAM1 on astrocytes.

**Conclusions:**

The behavior of ex-Th17 cells in EAE lesions in vivo resembles Th1 rather than Th17 cells, underlining that their change in cytokine production is associated with functional phenotype alterations of these cells.

**Supplementary Information:**

The online version contains supplementary material available at 10.1186/s12974-020-02021-x.

## Background

Adoptive transfer experimental autoimmune encephalomyelitis (EAE), in which myelin oligodendrocyte glycoprotein (MOG)-specific T cells are differentiated in vitro and subsequently transferred to recipient mice to induce EAE, is a common animal model to investigate T cell-driven neuroinflammation [1]. The protocols of T helper (Th)1 and Th17 differentiation in vitro as well as transfer EAE induction, however, are highly variable between different laboratories. This has contributed to contradicting results and controversy about the roles of Th1 and Th17 cells in EAE [2–7]. Th1 cells were originally thought to be the main pathogenic cells in EAE and multiple sclerosis (MS) [8–10]. The discovery of interleukin (IL)-23 as a critical cytokine for autoimmune neuroinflammation marked a turning point [3, 11], shifting the interest of research toward IL-17-secreting Th17 cells [12–14]. Th17 cells are a highly plastic T helper cell subset and contribute to both protective immunity and autoimmunity [15, 16]. Th17 cells have been shown to enter the central nervous system (CNS) via mechanisms different from Th1 cells causing diverse pathological and clinical phenotypes [17–19]. In recent years, however, it was increasingly recognized that the distinction between the Th1 and Th17 subsets in the context of neuroinflammation is not as clear as previously thought [20]. It has been shown that encephalitogenic Th17 cells can display pathogenic features previously attributed to Th1 cells [21] and vice versa [22]. Moreover, fate-mapping experiments demonstrated that Th17 cells in the CNS co-express or exclusively express interferon (IFN)γ [23, 24]. This led to the conclusion that Th17 cells can switch to an IFNγ-producing, ex-Th17 phenotype [25–27]. The molecular mechanisms of Th17 cell plasticity have been shown to be highly dependent on the tissue environment of the cells [26, 28–33], while the prerequisites shaping T cell phenotypes within the CNS tissue remain poorly understood. On the other hand, it has been shown that astrocytes as CNS-resident cells respond differentially to Th1 or Th17 presence during exposure and infiltration [34]. Astrocytes, as the primary cell type in the CNS, serve many neurotrophic and neuroprotective functions [35]. Moreover, they are the major source of extracellular matrix as well as adhesion molecules in the CNS [36].

Reactive astrocytosis (a potent and protective mechanism to seal off, e.g., blood-brain barrier (BBB) disruption) accompanies most neurological diseases [37]. Here, reactive astrocytes upregulate a number of integral molecules for the interaction with the peripheral immune system including excitatory amino acid transporters like GLAST or Glt1, as well as adhesion molecules like I-CAM or V-CAM [38, 39]. This subset of astrocytes serves as a liaison between the CNS and the peripheral immune system, especially at the BBB [40, 41]. In our study, we assessed the role of different T cell differentiation and transfer protocols for EAE disease development and investigated the functional dynamics of encephalitogenic T cells within the inflamed CNS tissue as well as their interplay with astrocytes.

## Methods

### Mice

IL-17 reporter mice (B6.IL17A-EGFP.acRFP x 2d2) were obtained by crossbreeding IL17A-EGFP mice (originally obtained from The Jackson Laboratory, JAX#018472) with B6.acRFP [42] and B6.2D2 [43] mice. 2d2.RFP mice were obtained by crossbreeding B6.acRFP [42] and B6.2D2 [43] mice. IL17A-EGFP mice carry an IRES-EGFP-SV40-polyA signal sequence cassette after the stop codon of the *Il17a* gene which results in the expression of enhanced green fluorescent protein (eGFP) as a marker of *Il17a* activity. B6.2d2 mice express a MOG_35-55_ peptide-specific T cell receptor [43] and B6.acRFP mice carry a tandem-dimer red fluorescent protein (tdRFP) cassette under transcriptional control of the ROSA26 locus resulting in ubiquitous expression of tdRFP [42]. EAE recipient mice were Rag 2^−/−^ mice [44] bred in-house. All animal experiments were approved by local authorities and conducted according to the German Animal Protection Law for care and use of experimental animals.

### T cell culture

Naïve CD4^+^ CD62L^+^ cells were isolated and MACS-sorted from spleens of donor mice (6–12 weeks old) with a purity of > 97% of total cells. Murine Th17 cell differentiation was achieved by adding 2 μg/ml αCD3, 3 ng/ml hTGF-β, 20 ng/ml IL-6, and 20 ng/ml IL-23 to culture medium. Irradiated antigen presenting cells (APCs) were used for initial stimulation in a 1:10 ratio. Cells were split with 50 U/ml IL-2 and 5–10 ng/ml IL-23 on days 3 and 5. Cells were restimulated with irradiated APCs in a 1:5 ratio on day 7 and harvested on day 10. Cytokine production was assessed using flow cytometry and cells that produced > 30% of IL-17 were used for experiments. Th1 differentiation was achieved by adding 2 μg/ml αCD3, 50 ng/ml IL-12, 25 ng/ml IL-18, and 10 μg/ml αIL-4. After 2 and 4 days of culture, T cells were split with 100 U/ml IL-2. Cells were harvested after 5 days of culture. Cytokine production was assessed using flow cytometry and cells that produced > 30% of IFNγ were used for experiments.

### Experimental autoimmune encephalomyelitis

To induce transfer experimental autoimmune encephalomyelitis (EAE), Rag 2^−/−^ mice were used as recipient mice. Cells were harvested, counted, and washed in PBS with calcium and magnesium (PBS(+)) three times. T cells in 200 μl of PBS(+) were transferred by intravenous (i.v.) injection into the tail vein. Following induction of EAE, clinical symptoms and weight were monitored daily starting on day 10. Clinical symptoms were translated to a clinical score from 0 to 5 as follows: Typical EAE: 0, no detectable signs of EAE; 0.5, tail weakness; 1, complete tail paralysis; 1.5, impaired righting reflex; 2, partial hind limb paralysis; 2.5, unilateral complete hind limb paralysis; 3, complete bilateral hind limb paralysis; 3.5, complete hind limb paralysis and partial forelimb paralysis; 4, total paralysis of forelimbs and hind limbs; and 5, death. Atypical EAE: 0, no detectable signs of EAE; 1, mild ataxia; 2, ataxia; 3, severe ataxia; 4, moribund; 5, death.

### Intravital two-photon laser scanning microscopy

Intravital two-photon laser scanning microscopy (TPLSM) was performed as described previously [16, 45, 46]. Briefly, excitation at 850 nm produced by a Ti:Sa laser was used to image the green channel and 1110 nm generated by an optical parametric oscillator (OPO) pumped by the Ti:Sa laser was used to image the red channel. A dichroic mirror (560DCXR) with filters at 525/50 for green and 593/40 for red was used to separate the fluorescence signals. Imaging was performed with a voxel size of 0.59 μm × 0.59 μm (sides) × 2 μm (depth). The scanner was set at 400 Hz, which corresponds to one image taken every 1.28 s. The stack was 300 × 300 × 72 μm. Image analysis was performed using Imaris software. Videos were cropped to 20 min and noise reduction was achieved using the software’s medium filter. Cell tracks were created using the Imaris tracking tool and manually corrected for accuracy. Track correction and verification was performed manually with 3D rotation.

### Astrocyte cultures

Astrocyte cultures were performed as previously described [47]. In brief, p0-p1 C57BL/6 brains were isolated and the hippocampus was stripped from the cortex. The cortex was digested in 1% DNase and 0.5% trypsin for 10 min at 37 °C. For homogenizing, tissue was sucked through two small glass pipettes and poured over a 70 μm mesh. Cells were seeded in DMEMC (DMEM with 1% Pen/Strep, 10% fetal bovine serum, 2 mM l-glutamine). Astrocytic cultures were inflamed at day 8. Cultures were harvested 24 h later by scraping for mRNA isolation.

### mRNA isolation and cDNA synthesis

For RNA preparation and cDNA synthesis, cells were processed according to the manufacturer’s instructions using the RNeasy Mini Kit (QIAGEN, Cat No: 74106). Concentrations were measured with a NanoDrop 2000c. For cDNA synthesis, 1 μg of RNA was mixed with random hexamers. Further, 5 mM MgCl2, 10× RT-Buffer, 0.01 M DTT, 1 mM dNTPs Mix, 0.8 U RNaseOUT, and 5 U Superscript III RT were added. The mix was incubated for 10 min at 25 °C, 50 min at 50 °C, and 5 min at 85 °C for cDNA synthesis.

For qPCR runs, 4 μl DNA were mixed with 10 μl SYBR Green, forward and reverse primer for V-CAM1 (AGACTACACTGATGAAGAA; GAGGCAAACAAGAGATTT), 200 nm for I-CAM1 (ACTGGACTATAATCATTC; CCTTCTGTAACTTGTATA) for Rps29 (CAAATACGGGCTGAACAT; GTCGCTTAGTCCAACTTAA), and filled up to 20 μl with water. qPCR analysis was performed in triplicate using a Bio-Rad CFX Connect cycler.

### EAE work-up and flow cytometry

Prior to CNS dissection, mice were perfused with PBS(−). The CNS was homogenized and digested with collagenase (5 mg/μl), collagenase/dispase (1000 U/μl), and DNase (1 mg/ml) for 30 min at 37 °C. The pellet was resuspended in either 30% percoll for T cell isolation or 20% percoll for astrocyte isolation. The upper phase was carefully layered on top of either 70% percoll (T cells) or 40% percoll (astrocytes). After continuous centrifugation (750 g, 30 min, room temperature, no break), the interphase was isolated.

For FACS analysis, cell pellets were stained for target antigens diluted in PBS (CD4 biotin, l/d V450 1:1000, GLAST APC 1:20, CD54 Biotin 1:200, Streptavidin PerCP 1:600, CD106 PeCy7 1:500). For fixation and subsequent intracellular staining, cells were incubated in 2% PFA for 20 min at 4 °C in the dark. All subsequent centrifugation steps were performed at 1000 g for 5 min at 4 °C. For FC-blocking, 70 μl Fc-blocking solution (αCD16/αCD32, 1:70 in Saponine buffer/perm buffer) was added and incubated for 10 min at 4 °C in the dark. For intracellular staining, intracellular target antigens diluted in Saponine buffer were added (IFNγ-AF700 1:200, IL17 FITC 1:400). Incubation occurred for 20 min at 4 °C in the dark. The cells were acquired at a FACS Canto II.

### Statistics

All data was analyzed using GraphPad Prism 6 software. Data are show as mean ± standard error of the mean (SEM) unless stated otherwise. Statistical analysis was performed as parametric (Student’s *t* test or ANOVA) or non-parametric (Mann-Whitney *U* test or Kruskal-Wallis test) depending on passing Shapiro-Wilk normality test. Significance was set at *p < 0.05.

## Results

### EAE courses are highly dependent on in vitro differentiation and transfer protocols

**Fig. 1:**
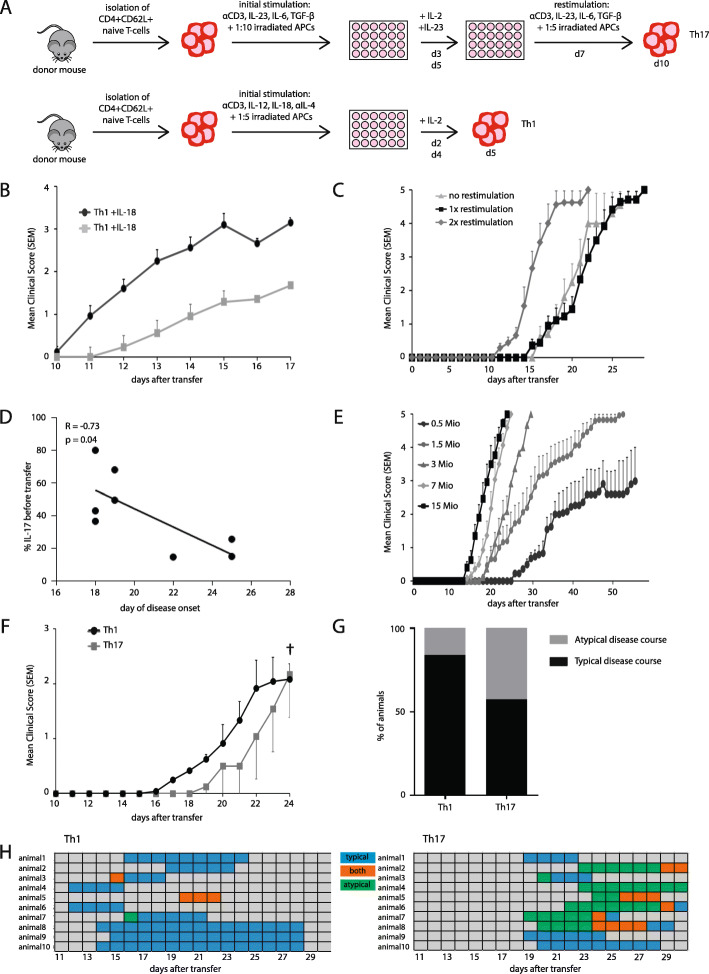
EAE courses depend on T-cell differentiation and transfer protocols. **a** Experimental protocols to skew naïve CD4^+^CD62L^+^ toward Th17 or Th1 cells. **b** IL-18 increases pathogenicity of Th1 cells. Animals were injected with Th1 cells differentiated with (black line) or without IL-18 (grey line). *n* = 12 animals per group. Mean clinical score and SEM shown. **c** The onset of disease is dependent on the restimulation of Th17 cells, whereas the clinical course remains unchanged. Mice received 10 mio Th17 cells without restimulation, or were restimulated once or twice. Group size 5–9 animals per group. Mean clinical score and SEM shown. **d** IL-17 expression levels correlate with the onset of disease symptoms. Data from eight independent EAEs shown. Day of disease onset marks day of first mouse showing EAE symptoms. IL-17 production was measured on the day of transfer using flow cytometry. Line marks linear regression; Spearman’s r is provided. **e** Disease onset and severity is dependent on the amount of transferred Th17 cells. *n* = 15 animals per group. Mean clinical score and SEM shown. **f** Disease courses dependent on Th1 or Th17 application. One representative experiment shown, *n* = 6 animals per group. † EAE had to be terminated due to animal protection regulations (including weight loss and overall appearance); normal disease course in this model. **g** Quantification of typical and atypical disease courses upon Th1 or Th17 application. Data from at least four independent experiments with group sizes of 25–40 animals. **h** Representative examples of disease courses of individual mice

In order to assess how changes in protocol influence the onset and course of EAE, we analyzed protocols from the literature to identify aspects with high variability between different laboratories (Table 1). We identified the in vitro differentiation protocol, especially cytokine addition and restimulation, as well as the amount of transferred cells in adoptive transfer EAE using Rag 2^−/−^ recipient mice as possible modulating factors for further assessment. Th1 and Th17 cells were differentiated from naïve CD4^+^CD62L^+^ T cells isolated from spleens of 2d2 donor mice (Fig. 1a).

**Table 1:** Representative transfer EAE induction protocols in the literature

Publication	Genotype of recipient mice	Age of recipient mice (weeks)	Cell count (mio.)	T cell subset	Route	T-cell donor pre-immunization	Organ
Williams et al. 2020 [48]	C57BL/6J	8-9	10	Th1	Retro-orbital i.v.	50 μg MOG (aa35-55) in IFA with 500 μg/ml *M. tuberculosis*	Spleen
Stromnes et al. 2008 [4]	Sublet. irradiated C3HeB/Fej mice	Unknown	20	Th1 Th17	i.p.	rMOG-immunized mice for 3 days with rMOG (25 μg/ml) or MOG peptides	Spleen
Domingues et al. 2010 [19]	Rag2^−/−^	Unknown	5-10	Th1 Th17 both	Tail vein i.v.	No, but 2d2 or OSE genotype	Spleen
Kroenke et al. 2010 [49]	C57BL/6J	8-12	5-6	Th1	i.p.	s.c. 100 μg MOG (aa35–55) in complete FA	LN
Rothhammer et al. 2011 [17]	Rag1^−/−^	Unknown	2 of cytokine positive cells	Th1 Th17	i.v.	No, but 2d2	Spleen, LN
Jäger et al. 2009 [18]	C57BL/6J	7-10	3-5	Th1 Th17	i.v.	No, but 2d2	Spleen, LN
Our protocol	Rag2^−/−^	8-12	5-10	Th1 Th17	Tail vein i.v.	No, but 2d2	Spleen, LN

*Th* T helper cell. *i*.*v*. intravenous, *MOG* myelin oligodendrocyte glycoprotein, *IFA* incomplete Freund’s adjuvant, *i*.*p*. intraperitoneal, *FA* Freund’s adjuvant, *LN* lymph node

A major difference in differentiation protocols of Th1 cells in literature is the use of IL-18 (Table 2), a pro-inflammatory cytokine that has been shown to enhance the IL-12-driven Th1 immune response [50]. To investigate to what extent IL-18 added to cell culture can influence the later EAE course, we performed Th1 differentiation with or without addition of 25 ng/ml of IL-18. When transferred into recipient mice, Th1 cells differentiated with IL-18 caused earlier onset of disease as well as stronger disease symptoms (Fig. 1b). The Th17 cell differentiation protocol was modulated by varying the time cells were cultured in vitro and the number of restimulations with APCs that were performed during culture. Th17-skewed cells were either cultured without restimulation for 5 days in vitro, with one restimulation after 7 days of culture or with a second restimulation after 14 days of culture before the transfer into Rag 2^−/−^ recipient mice. A second restimulation, and therefore longer cultivation in vitro, led to an earlier disease onset compared to mice which received Th17 cells that had not been restimulated, or had been restimulated only once. The disease course, however, was similar between the different groups (Fig. 1c).

**Table 2:** Differential cytokine applications for Th1 and Th17 induction

Publication	APC (10^6^)	MOG (aa35-55)	Anti-CD28	Anti-CD3	IL-12	IL-2	IL-18	Anti-IL-4	rmIFNγ	IL-23	hTGF-β	IL-6	Anti-IFNγ	Restimulation in vitro
Williams et al. 2020 [48]	5	10 μg/ml	/	/	10 U/ml (Th1)	10 U/ml (Th1)	/	/	/	/	/	/	/	Yes
Stromnes et al. 2008 [4]	/	/	/	/	10 ng/ml (Th1)	/	/	/	/	10 ng/ml (Th17)	/	/	/	No
Domingues et al. 2010 [19]	/	20 μg/ml (MOG aa1-125)	/	/	10 ng/ml (Th1)	10 ng/ml at day 3 (Th1)	25 ng/ml (Th1)	10 μg/ml (Th1) (Th17)	/	10 ng/ml (Th17)	5 ng/ml (Th17)	20 ng/ml (Th17)	/	Yes
Kroenke et al. 2010 [49]		4 days with MOG	/	/	5 ng/ml (Th1)	/	/	10 μg/ml (Th1)	2 ng/ml (Th1)	/	/	/	/	No
Rothhammer et al. 2011 [17]	Irradiated 1:5 ratio	20 μg/ml (Th1) (Th17)	2 μg/ml plate-bound (Th1) (Th17)	4 μg/ml plate-bound (Th1) (Th17)	10 ng/ml (Th1)	/	/	10 μg/ml (Th1) (Th17)	/	25 ng/ml (Th17)	2 ng/ml (Th17)	20 ng/ml (Th17)	/	Yes
Jäger et al. 2009 [18]	Irradiated 1:5 ratio	20 μg/ml	/	2.5 μg/ml (Th1) (Th17)	10 ng/ml (Th1)	/	/	20 μg/ml (Th1) (Th17)	/	/	3 ng/ml (Th17)	30 ng/ml (Th17)	20 μg/ml (Th17)	Yes
Our protocol	Irradiated 1:5 (Th1) ratio 1:10 (Th17)	/	/	2 μg/ml (Th1) (Th17)	5 ng/ml (Th1)	/	25 ng/ml (Th1)	10 μg/ml (Th1) (Th17)	**/**	20 ng/ml (Th17)	3 ng/ml (Th17)	20 ng/ml (Th17)	10 μg/ml (Th17)	Yes

*MOG* myelin oligodendrocyte glycoprotein

In addition, the role of IL-17 for disease development was evaluated. IL-17 production of CD4^+^ cells was measured by flow cytometry prior to transfer into recipient mice and compared to the day of disease onset defined as the day on which the first mouse showed EAE symptoms. IL-17 production and day of disease onset were significantly correlated (Fig. 1d). Notably, mice which received Th17 cells with a higher IL-17 production on the day of transfer showed symptoms of EAE significantly earlier.

Next, to investigate how the EAE course depends on the number of transferred cells, transfer EAE was induced by different amounts of Th17-skewed cells. Th17 cells were differentiated in vitro following a protocol with one restimulation and subsequently transferred to recipient mice. Mice received cells on the same day from the same culture to exclude confounding factors in the in vitro differentiation. With 15 or 7 million cells received, mice showed similar disease courses characterized by a fast acceleration of disease symptoms reaching a maximum score within a few days. When mice received 3 million Th17 cells, they showed a similarly fast disease course, but the onset was slightly delayed. Further, 1.5 million Th17 cells led to a similar day of onset as with 3 million transferred cells, but a slower and thereby prolonged disease course compared to higher cell numbers. Moreover, when receiving only 0.5 million Th17 cells on the day of transfer, mice showed an even later onset and a more prolonged disease course as well as a lower maximum clinical score (Fig. 1e).

Subsequently, the difference between Th1- and Th17-induced transfer EAE was directly compared by transferring 10 million Th1 or Th17 cells into recipient mice. While Th1-induced EAE showed an earlier day of onset and earlier worsening of symptoms compared to Th17-induced EAE, mice reached similar clinical scores by the end of the observation period (Fig. 1f). Previously, Th1 EAE has been associated with “typical” symptoms (i.e., ascending paralysis), while Th17 EAE was associated with “atypical” symptoms (i.e., brainstem and cerebellar symptoms manifesting as ataxia) [19]. In our study, there was indeed a clear trend toward more cases of atypical EAE symptoms in Th17-induced transfer EAE, although atypical symptoms also occurred in some Th1 mice (Fig. 1g). Interestingly, a closer examination of individual disease courses revealed that mice that had received Th17 cells frequently showed atypical EAE symptoms at the beginning of disease, which later transitioned to typical EAE symptoms. This phenomenon was not observed in Th1-induced EAE (Fig. 1h).

### Encephalitogenic Th17 cells acquire an ex-Th17 IFNγ-producing phenotype in transfer EAE

**Fig. 2:**
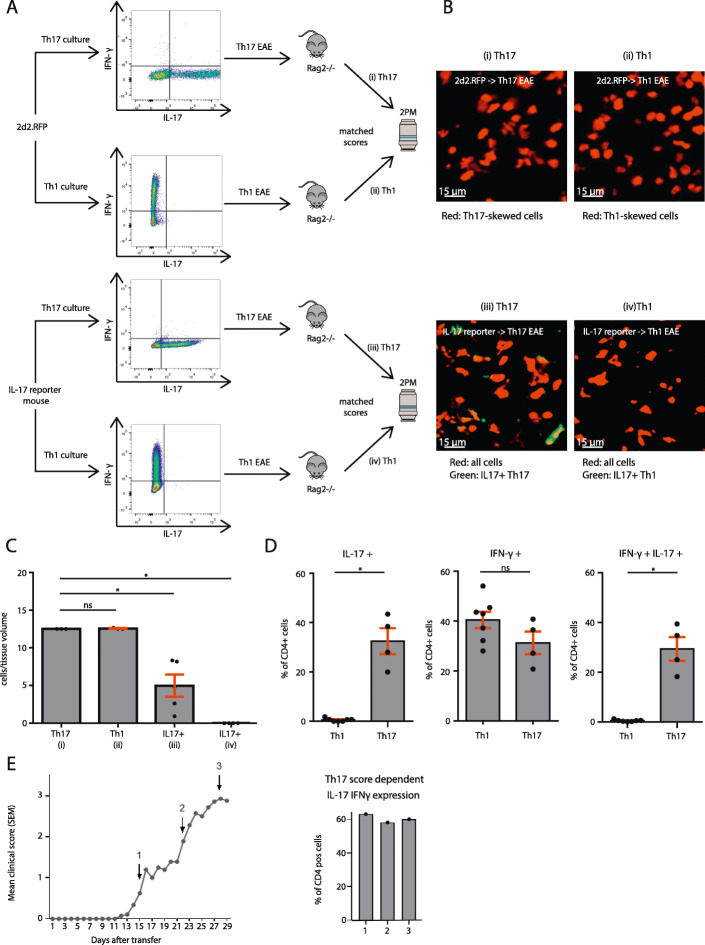
Encephalitogenic Th17 cells acquire an ex-Th17 IFN-γ-producing phenotype in vivo. **a** Experimental setup for TPLSM experiments. Naïve T cells from 2d2.RFP or IL-17 reporter mice were skewed to Th1 or Th17 cells. Flow cytometry was performed to verify appropriate cytokine profile. Staining was performed using antibody staining of PFA-fixed cells. Cells were then transferred to recipient mice. **b** Representative snapshots from TPLSM videos of different conditions. (i) transfer of 10 million 2d2 Th17-skewed cells, (ii) transfer of 10 million 2d2 Th1-skewed cells, (iii) transfer of 10 million IL-17-reporter Th17-skewed cells, (iv) transfer of 10 million IL-17-reporter Th1-skewed cells. IL-17 reporter mice express eGFP as a marker of *Il17a* activity, a MOG-specific T cell receptor, and ubiquitously express tdRFP. 2d2.RFP mice express a MOG-specific T-cell receptor and ubiquitously express tdRFP. **c** Quantification of cells per tissue volume (10^6^ μm^3^) in videos from different conditions described in (**b**). Data from at least 3 different mice per condition shown. **d** Quantification of single cell, living CD4^+^ cells after either Th1 or Th17 application. Animal scores were above 2. *p < 0.05, **p < 0.01 *n* = 4–7 animals per group. **e** Representative disease of 14 mice from two independent experiments. IFNγ expression (right) during Th17-induced disease (left). Expression levels of IFNγ in ex-Th17 were measured when mice displayed clinical scores of 1, 2, and 3. Expression levels have already reached their final level at the onset of disease and remain stable over its course. *n* = 3

Visualization of T cell infiltration within CNS lesions assessed by in vivo two-photon microscopy is a powerful tool to unravel inflammatory processes directly within the tissue [51, 52]. Therefore, Th1 and Th17 cells from 2d2.RFP or IL-17 reporter mice were differentiated following the protocol shown in Fig. 1a and transferred to Rag 2^−/−^ recipient mice (Fig. 2a). Cells were monitored in the inflamed CNS at the peak of disease using TPLSM (Fig. 2b, Sup. Figure 1A). We found similar numbers of Th17- and Th1-skewed cells in the CNS. In addition, we found significantly lower levels of IL-17-producing Th17-skewed cells and as expected, no IL-17-producing Th1-cells (Fig. 2c). When animals reached a clinical score of two, T cells were isolated from the CNS and their cytokine profile was analyzed using flow cytometry. Th1 cells showed a stable IFNγ production and did not express IL-17, while Th17 started to co-express IFNγ in addition to their IL-17 production. IFNγ expression levels in Th17 cells were comparable to levels in Th1 cells, underlining the acquisition of an ex-Th17 phenotype (Fig. 2d, Sup. Figure 1B and C). The IFNγ expression levels in Th17 cells reached their final expression level already at the beginning of symptoms and remained stable over time (Fig. 2e).

### Ex-Th17 cells show functional dynamics similar to Th1 cells but different from Th17 cells in the inflamed CNS tissue

**Fig. 3:**
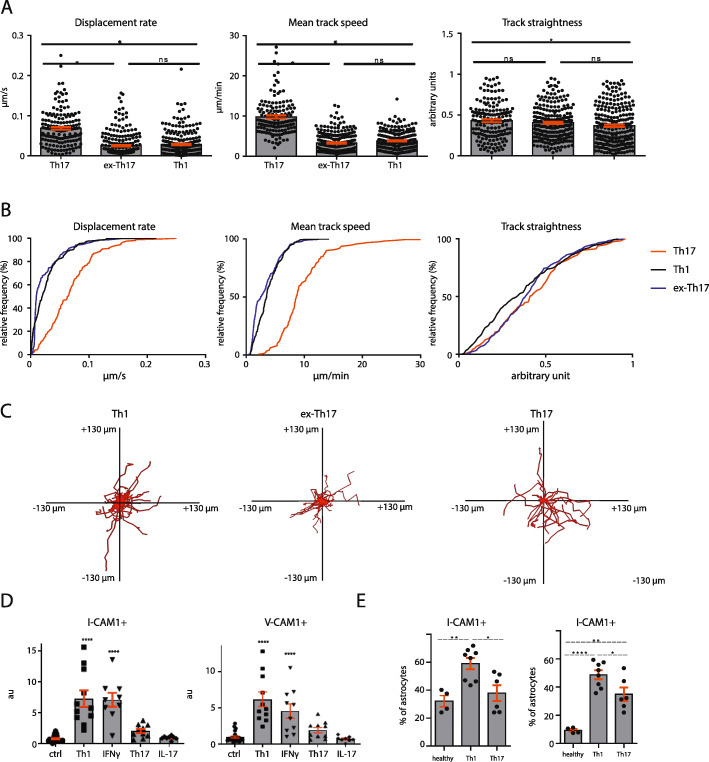
Ex-Th17 cells resemble Th1 cells in terms of motility in vivo and differentially induce I-CAM1 and V-CAM1 expression on astrocytes. **a** Motility parameters of Th17, ex-Th17, and Th1 cells in vivo. Th17: IL-17-producing cells as indicated by IL-17 reporter mouse. Ex-Th17: 2d2.RFP Th17-skewed cells. Th1: 2d2.RFP Th1-skewed cells. Displacement rate (displacement length divided by track duration), mean track speed and track straightness shown for different cell types. Data from at least 3 independent animals per group. *p < 0.05, one-way ANOVA. **b** Relative frequency distribution of displacement rate, mean track speed and track straightness of IL-17 producing Th17 cells (red), Th1 cells (black), and ex-Th17 cells (blue). **c** Superimposed tracks of Th1, ex-Th17, and Th1 cells. One representative experiment per condition shown. Track duration 20 min. Track numbers shown: Th17 54 tracks, ex-Th17 81 tracks, Th1 81 tracks. **d** mRNA quantification of astrocytes in vitro after exposure to either Th1 or Th17 cells or their signature cytokines IFNγ or IL-17. Data from 8 to 11 independent cultures. ****p < 0.0001. **e** I-CAM1 and V-CAM1 protein expression on cerebral astrocytes during Th1- or Th17-induced EAE of two independent experiments. EAE scores are above 2. Four to nine animals analyzed. *p < 0.05

We subsequently assessed the motility parameters of Th17, ex-Th17, and Th1 cells in the CNS tissue. Ex-Th17 cells were defined as Th17-differentiated cells which showed no detectable IFNγ production on the day of transfer, but started producing IFNγ after infiltrating into the CNS as measured by flow cytometry. We found significant differences between Th17 and Th1 cells in displacement rate, mean track speed, and track straightness indicating different functions exerted by the cell types within the tissue. Th1 cells moved slower, less straight, and with a lower displacement rate than Th17 cells. Interestingly, ex-Th17 cells showed a motility significantly different from Th17 cells, but similar to Th1 cells (Fig. 3a–c, Sup. Video 1, 2, 3).

### IFNγ-producing Th1 cells selectively induce I-CAM1 and V-CAM1 expression on astrocytes

Differences in neuroinflammatory responses within the CNS have been proposed to be mediated by T cell subtype-specific interactions with brain-resident cells. Astrocytes forming tight junctions with the vasculature, the main infiltration route, are potentially the first brain-resident cells to interact with infiltrating T cells [53, 54]. This last step of lymphocyte migration into the CNS after crossing the endothelial cell barrier (i.e., migration from the perivascular space into the CNS parenchyma) has so far been less well-characterized, although astrocytes are clearly implicated in this process [55]. This interaction may be adhesion molecule-mediated as astrocytes are the major CNS source producing among others I-CAM1 and neural cell adhesion molecule (N-CAM), as well as V-CAM1 [36, 38]. Therefore, we investigated the expression of adhesion molecules on astrocytes in vitro and upon EAE induction with different Th subsets. Indeed, co-culture of cortical astrocytes with either Th1 or Th17 cells or their signature cytokines and subsequent RT-PCR showed that Th1 and IFNγ selectively induce intracellular and vascular cell adhesion molecule (I-CAM1 and V-CAM1) transcription on this subset of astrocytes (Fig. 3d). Neither Th17 cells nor IL-17 were able to induce a significant upregulation of transcription. In vivo, both Th1 and Th17 cells were able to induce high levels of V-CAM1 expression on astrocytes (Fig. 3e, Sup. Figure 1D), whereas I-CAM1 expression was exclusively induced by Th1 cells, both correlating well with the RT-PCR data.

## Discussion

EAE is the most commonly used animal model to investigate MS. It shares key immunopathological and neuropathological features such as inflammation and resolution of inflammation, demyelination, and gliosis with the human disease [56] and pivotal contributions to MS research have been made using the EAE model. However, EAE protocols, especially for adoptive transfer EAE, are highly variable between laboratories [4, 5, 17–19, 48, 49]. In our study, we showed how changes in the EAE protocol can significantly influence the dynamics of the disease. In the context of cytokine stimulation and Th1 differentiation in vitro, especially the role of IL-12 for development of EAE has been extensively discussed. While some groups have shown that IL-12 stimulation is sufficient to induce encephalitogenicity in CD4^+^ cells [7, 22], these findings have been challenged by the ability to induce EAE in mice deficient in IL-12 [57] and the fact that donor T cells in these studies derived from wild-type mice that could have been exposed to endogenous IL-23 in the donor mouse [22]. Still, IL-12 is a common feature of all Th1 differentiation protocols analyzed and was therefore not looked at in detail in this study. In contrast, the IFNγ-inducing factor IL-18 [50]—having been shown to play a role in both MS [58] and EAE [59, 60] and to contribute to IFNγ-induction in Th1 cells [50, 61]—was only used in some Th1 differentiation protocols. We therefore investigated the influence of IL-18 during the differentiation of Th1 cells on the eventual EAE course. We found increased pathogenicity of Th1 cells differentiated in the presence of IL-18 underlining the importance of IL-18 for Th1 differentiation. In addition, we identified restimulation of Th17 cells in vitro, IL-17 production on the day of transfer, and number of cells transferred as influencing variables on the disease course. The T cell subset transferred to recipient mice played a role only during the onset of the disease. Mice having received Th17 cells showed a later onset and slower acceleration of disease severity, but reached similar EAE scores when compared to mice having received Th1 cells. This delay in disease activity could be explained by a possible need for Th17-skewed cells to acquire an IFNγ-producing phenotype in order to show a similar pathogenicity as Th1-skewed cells. This is also in accordance with our observation that mice which received Th17 cells showed an atypical EAE phenotype in the beginning of the disease and later switched to typical EAE symptoms and could be confirmed by our flow cytometry data showing high IL-17 and IFNγ co-expression at the beginning of the disease.

Th17 cells are a highly plastic T cell subset with the ability to acquire features originally attributed to other T cell subsets depending on the environmental context. In our study, we confirmed that T cells skewed toward Th17 cells in vitro acquire an IFNγ-producing Th1-like phenotype in vivo based on flow cytometry [13, 24]. In addition, we were able to show for the first time that IFNγ-producing ex-Th17 cells functionally resemble Th1 rather than Th17 cells based on the functional dynamics of the cells in the CNS. Using intravital TPLSM, we were able to monitor the different T cell subsets in the CNS in vivo. In this set-up, Th1 cells and ex-Th17 cells showed a motility behavior different from Th17 cells. They moved slower, less straight, and with a lower displacement rate. These parameters are indicative of an increased cell-cell contact and higher pathogenicity [52]. Previous reports on Th1 and Th17 motility within the CNS showing a different behavior of the cell types have used a different EAE paradigm and monitored cells in the spinal cord close to blood vessels and are therefore not comparable to our experimental setup [62]. Our results, however, contribute to previous reports claiming ex-Th17 cells unite various detrimental properties highlighting their role for pathogenicity in autoimmunity [26]. Moreover, it shows that the dogmatic distinction between Th1- and Th17-induced EAE is not useful in the context of neuroinflammation as Th17 cells are able to exert functions similar to Th1 cells in the CNS.

O’Connor et al. proposed that Th1 cells have the ability to facilitate the entry of Th17 cells to the CNS in EAE and identified IFNγ as a critical cytokine for this process [2]. In vivo imaging of the BBB recognized continuous coverage of the vasculature by astrocytic end-feet [63] potentially allowing for direct interaction with infiltrating Th1 and Th17 cells. As mentioned earlier, astrocytes are the major source of adhesion molecules in the brain. This adhesion molecule expression is known to be essential for inflammation development in the astrocytic parenchyma [64]. I-CAM1 expression levels are low on astrocytes under homeostatic conditions but will be upregulated in vitro by IFNγ, leading to the generation of neurotoxic astrocytes [65, 66]. Our data show that in vivo Th1 are able to upregulate I-CAM1 expression on astrocytes. V-CAM1 interacts with VLA4 and integrin α4β7 expressed on circulating immune cells. In the homeostatic brain, V-CAM1 is almost exclusively expressed by astrocytes in the CNS [67]. Recently, Williams et al. described a Th17- and region-dependent increase of V-CAM1 in the brainstem and spinal cord [48]. Based on our data, we cannot confirm a Th17-dependent effect in the cortex. However, in this region, the induction of V-CAM1 appears to be dependent on Th1 cells. This is in line with observations showing that astrocytes are functionally and morphologically diverse from the moment they develop allowing for region-dependent functionality and interaction partners [68, 69]. In vitro, Th17 cells are not able to induce V-CAM1 expression and only upon gaining an IFNγ-producing ex-Th17 phenotype in vivo did we observe V-CAM1 induction on astrocytes.

## Conclusion

In conclusion, we show that in vitro differentiated Th17 cells are able to acquire an IFNγ-producing ex-Th17 phenotype in EAE. These ex-Th17 cells show a behavior resembling Th1 cells rather than Th17 cells in EAE lesions in vivo. From this, we conclude that Th17 cells do not only alter their cytokine production when switching to an ex-Th17 phenotype but that the conversion is accompanied by a functional phenotype alteration of the cell with a potential increase in pathogenicity.

## Supplementary Information

**Additional file 1:** **Supplementary Figure 1** A) Representative snapshots from TPLSM videos of IL17-reporter mice. Red: all cells, green: IL-17+ cells. Scale bar: 10 μm. B) Exemplary gating strategy for Fig. 2d. Cells were gated for single cells, losing doublets. In a next step, the living cells were analyzed and the CD4+ population was analyzed in more depth. Within this population we looked for IL-17 and IFNγ single and double positive cells. C) Exemplary flow cytometry plots for IL-17 and IFNγ production of cells isolated after Th1 or Th17 cell application. D) Exemplary flow cytometry plots for astrocytes after Th1 application analyzing I-CAM1 and V-CAM1 positivity.

**Additional file 2:** **Supplementary Video 1** Two-photon live imaging of 2d2.RFP ex-Th17 cells. EAE was induced in Rag2^−/−^ mice via transfer of Th17-skewed 2d2.RFP cells. Shown here is the original RFP (ex-Th17 cells) 3D image sequence, smoothened and 3D cropped using Imaris. Time is shown in h/min/s/ms.

**Additional file 3:** **Supplementary Video 2** Two-photon live imaging of 2d2.RFP Th1 cells. EAE was induced in Rag2^−/−^ mice via transfer of Th1-skewed 2d2.RFP cells. Shown here is the original RFP (Th1 cells) 3D image sequence, smoothened and 3D cropped using Imaris. Time is shown in h/min/s/ms.

**Additional file 4:** **Supplementary Video 3** Two-photon live imaging of IL-17 reporter Th17 cells. EAE was induced in Rag2^−/−^ mice via transfer of Th17-skewed IL-17 reporter cells. Shown here is the original RFP (all cells) and GFP (IL-17-producing Th17 cells) 3D image sequence, smoothened and 3D cropped using Imaris. Time is shown in h/min/s/ms.

## Data Availability

The datasets generated and analyzed during the current study are available from the corresponding author on reasonable request.

## Abbreviations

APC: Antigen-presenting cell
BBB: Blood-brain barrier
CNS: Central nervous system
EAE: Experimental autoimmune encephalomyelitis
eGFP: Enhanced green fluorescent protein
FA: Freund’s adjuvant
I-CAM: Intracellular adhesion molecule
IFA: Incomplete Freund’s adjuvant
IFN: Interferon
IL: Interleukin
i.p: Intraperitoneal
i.v.: Intravenous
LN: Lymph node
MOG: Myelin oligodendrocyte glycoprotein
MS: Multiple sclerosis
tdRFP: Tandem-dimer red fluorescent protein
Th: T helper cell
TPLSM: Two-photon laser scanning microscopy
V-CAM: Vascular cell adhesion molecule
