# Supplementary material for: Functional characteristics of Th1, Th17, and ex-Th17 cells in EAE revealed by intravital two-photon microscopy
Source: J Neuroinflammation. 2020 Nov 26;17:357. doi: 10.1186/s12974-020-02021-x (PMC7694901; doi:10.1186/s12974-020-02021-x)
Supplement: Supplementary file 1 — Additional file 1: Supplementary Figure 1 A) Representative snapshots from TPLSM videos of IL17-reporter mice. Red: all cells, green: IL-17+ cells. Scale bar: 10 μm. B) Exemplary gating strategy for Fig. 2d. Cells were gated for single cells, losing doublets. In a next step, the living cells were analyzed and the CD4+ population was analyzed in more depth. Within this population we looked for IL-17 and IFNγ single and double positive cells. C) Exemplary flow cytometry plots for IL-17 and IFNγ production of cells isolated after Th1 or Th17 cell application. D) Exemplary flow cytometry plots for astrocytes after Th1 application analyzing I-CAM1 and V-CAM1 positivity. [file 12974_2020_2021_MOESM1_ESM.pdf]

Functional characteristics of Th1, Th17 and ex-Th17 cells in EAE revealed by intravital two-photon microscopy

Julia Loos, Samantha Schmaul, Theresa Noll, Magdalena Paterka, Miriam Schillner, Julian T. Löffel, Frauke Zipp, Stefan Bittner

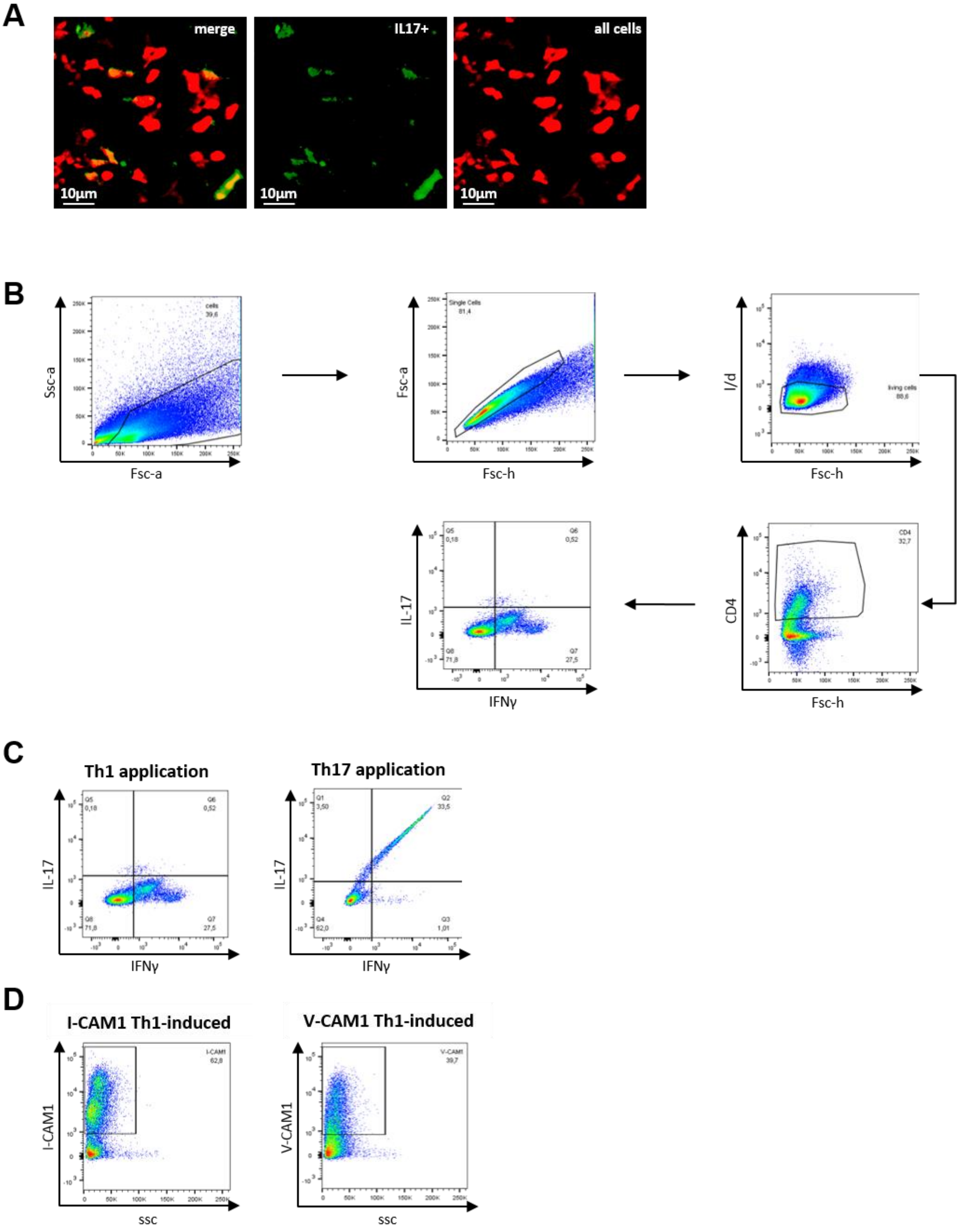

Supplementary Figure 1

A) Representative snapshots from TPLSM videos of IL17-reporter mice. Red: all cells, green: IL-17+ cells. Scale bar: 10 µm. B) Exemplary gating strategy for Figure 2D. Cells were gated for single cells, losing doublets. In a next step, the living cells were analyzed and the CD4+ population was analyzed in more depth. Within this population we looked for IL-17 and IFNγ single and double positive cells. C) Exemplary flow cytometry plots for IL-17 and IFNγ production of cells isolated after Th1 or Th17 cell application. D) Exemplary flow cytometry plots for astrocytes after Th1 application analyzing I-CAM1 and V-CAM1 positivity.

Supplementary Video 1 Two-photon live imaging of 2d2.RFP ex-Th17 cells

EAE was induced in Rag2$^{-/-}$ mice via transfer of Th17-skewed 2d2.RFP cells. Shown here is the original RFP (ex-Th17 cells) 3D image sequence, smoothened and 3D cropped using Imaris. Time is shown in h/min/s/ms.

Supplementary Video 2 Two-photon live imaging of 2d2.RFP Th1 cells

EAE was induced in Rag2$^{-/-}$ mice via transfer of Th1-skewed 2d2.RFP cells. Shown here is the original RFP (Th1 cells) 3D image sequence, smoothened and 3D cropped using Imaris. Time is shown in h/min/s/ms.

Supplementary Video 3 Two-photon live imaging of IL-17 reporter Th17 cells

EAE was induced in Rag2$^{-/-}$ mice via transfer of Th17-skewed IL-17 reporter cells. Shown here is the original RFP (all cells) and GFP (IL-17-producing Th17 cells) 3D image sequence, smoothened and 3D cropped using Imaris. Time is shown in h/min/s/ms.
